# The Concept of Missing Incidents in Persons with Dementia

**DOI:** 10.3390/healthcare3041121

**Published:** 2015-11-10

**Authors:** Meredeth Rowe, Amy Houston, Victor Molinari, Tatjana Bulat, Mary Elizabeth Bowen, Heather Spring, Sandra Mutolo, Barbara McKenzie

**Affiliations:** 1 College of Nursing, University of South Florida, 12901 Bruce B. Downs Blvd., Tampa, FL 33612, USA; E-Mail: prayerstudy@gmail.com; 2 Department of Psychology, Xavier University, Cincinnati, OH 45206, USA; E-Mail: Houstona1@xavier.edu; 3 School of Aging Studies, College of Behavioral and Community Sciences, University of South Florida, 13301 Bruce B. Downs Blvd., Tampa, FL 33612-3899, USA; E-Mail: vmolinari@usf.edu; 4 VISN 8 Patient Safety Center of Inquiry, James A. Haley Veterans Hospital, 8900 Grand Oak Circle, Tampa, FL 33637, USA; E-Mails: Tatjana.Bulat@va.gov (T.B.); Sandra.mutolo@va.gov (S.M.); Barbara.McKenzie@va.gov (B.M.); 5 Department of Health, 315 Sturzebecker Health Sciences Center, West Chester University of Pennsylvania, West Chester, PA 19383, USA; E-Mail: mbowen@wcupa.edu

**Keywords:** dementia, missing, wandering, lost, behavioral symptoms of dementia

## Abstract

Behavioral symptoms of dementia often present the greatest challenge for informal caregivers. One behavior, that is a constant concern for caregivers, is the person with dementia leaving a designated area such that their whereabouts become unknown to the caregiver or a missing incident. Based on an extensive literature review and published findings of their own research, members of the International Consortium on Wandering and Missing Incidents constructed a preliminary missing incidents model. Examining the evidence base, specific factors within each category of the model were further described, reviewed and modified until consensus was reached regarding the final model. The model begins to explain in particular the variety of antecedents that are related to missing incidents. The model presented in this paper is designed to be heuristic and may be used to stimulate discussion and the development of effective preventative and response strategies for missing incidents among persons with dementia.

## 1. Introduction

A missing incident for a person with dementia (PWD) is defined as an instance in which the PWD’s whereabouts are unknown to the caregiver and the individual is not in an expected location [1]. Previously, these incidents had been referred to as “wandering”, but over the past decade, a significant body of research conducted on both wandering and missing incidents suggests that these are conceptually distinct behavioral symptoms associated with dementia. Wandering has been defined as:

A syndrome of dementia-related locomotion behaviour having a frequent, repetitive, temporally-disordered and/or spatially-disoriented nature that is manifested in lapping, random and/or pacing patterns, some of which are associated with eloping, eloping attempts or getting lost unless accompanied [2].

**Figure 1:**
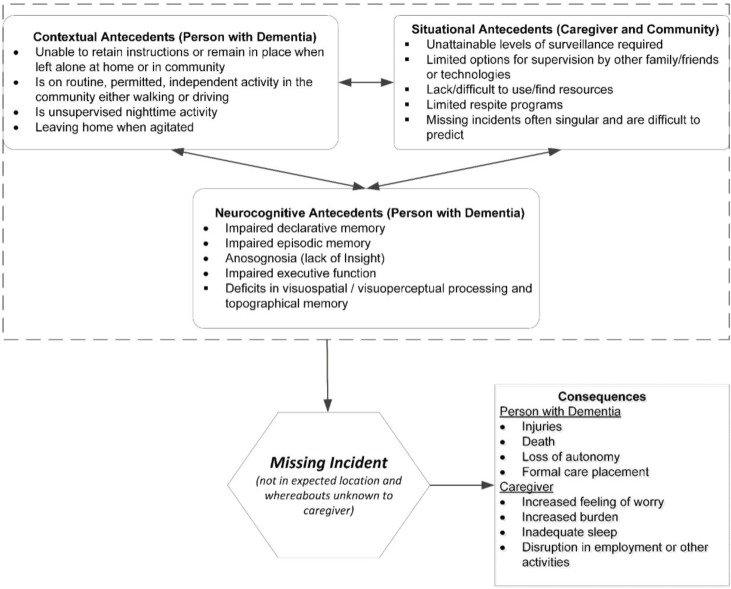
Missing Incident Conceptual Model.

In proposing a distinct definition for missing incidents, Rowe *et al*. (2011) [1] found that a missing incident differs from wandering behavior in terms of frequency (singular or isolated *vs*. regularly repeated activity, respectively), timing (unpredictable *vs*. occurring within predictable time frames), and nature (occurring within normally conducted activities *vs*. being temporally and spatially-disoriented.

Bringing clarity and definition to the concept of missing incidents is critical because it is likely that the majority of persons with dementia (PsWD) will have at least one missing incident during the course of their disease [3]. One longitudinal study reported the incidence for Veterans with dementia to be 0.46 incidents/year [4]. Although injuries and death are surprisingly rare [5], missing incidents have other consequences including earlier institutional placement for the PWD [6], high stress and increased vigilance for the caregiver and an increase in law enforcement and search resources for the community [4,7,8]. The purpose of this paper is to propose a unique conceptual model (see Figure 1) to stimulate discussion and future research to fully develop this concept and target interventions to prevent missing incidents or strategies to more effectively find the lost individual.

## 2. Methodology

The need for a distinct conceptual model of missing incidents involving PsWD was mandated by an expert panel of the International Consortium on Wandering and Missing Incidents, a multi-disciplinary association of experts on issues related to wandering and related concepts. During the ten years of its existence, members have remained current on these topics, conducted original research and published a number of articles on both wandering and missing incidents. From our combined research and experience, we became increasingly convinced that “wandering” and “missing incidents” were two distinct concepts, although we acknowledged that the former could sometimes lead to the latter. Therefore, we conducted an extensive literature review to identify applicable studies on the missing incidents and related topics. This search was conducted on the Medline and PsychInfo databases using combinations of the terms “missing/lost/wandering/elopement” with “Alzheimer’s disease/dementia cognitively-impaired”. Data-based articles were printed and used if the study focused on community-dwelling persons with dementia who were missing or found in the community, or caregivers were surveyed on the issue of missing incidents in their care recipient. The available literature was somewhat limited so all available articles were used in team discussions to formulate this provisional model.

Information from these studies and our own work was summarized by assigned team members and then reviewed and modified by all the other team members until a consensus on the model was reached. This model is considered a work in progress, and is intended to stimulate discussion and define needs of future research. The authors hope to guide the thinking about factors leading to and consequence of a missing incident, and believe that the model will prove heuristic in stimulating more research across the different domains of missing incidents.

## 3. Results and Discussion

### 3.1. Antecedents

In order for a missing incident to occur, we hypothesize that there is interplay among contextual, situational and neurocognitive antecedents that leads to the PWD leaving a safe situation and then going missing. The importance of each of these factors in preceding or facilitating a missing incident is discussed below.

#### 3.1.1. Contextual Antecedents

From a series of studies conducted by Consortium researchers, and from the literature review, there are a number of contextual antecedents that immediately precede the movement of the PWD from a safe setting to an unknown location. Researchers in an early study investigating this phenomenon categorized the circumstances as: being on a normal outing without returning, getting separated from the caregiver while in the community, and leaving the home when agitated [9]. These categories were further refined through a series of studies, and an inclusive set of circumstances was confirmed in the latest study [1]. The most crucial characteristic of all the contextual antecedents is a gap in supervision of the PWD [1,4,9,10,11].

The most common context in which this gap occurs is when the PWD is intentionally left alone and instructed to remain in a given place until the caregiver returns. This gap occurs when the dyad is together at home or in the community. Common examples include being left in one part of the home while the caregiver is occupied elsewhere or being left at the front of a store whilst the caregiver shops. In this context the intentional supervision gap is very short, often less than 10 minutes [4].

The second most common context for a missing incident occurs while a PWD is on a routine, permitted (by the caregiver), independent community activity. Most commonly, the PWD is on a walk in the neighborhood for exercise or enjoyment of some personal independence. These activities are almost always to familiar locations [12]. Usually, the PWD has independently conducted this journey on multiple occasions with a successful return [9]. The importance of this context is further emphasized in two studies of those who go missing while driving. Hunt, Brown, and Gilman reviewed 207 published reports of PsWD who became lost while driving and found that most become lost while driving independently to familiar places [13]. Consistent with the above findings, in an analysis of 157 cases of missing drivers, over half of had been engaged in a normally-conducted trip prior to getting lost [10].

Two other contextual antecedents consistently appear in the literature: leaving the home during the night when the PWD has arisen but the caregiver continues to sleep and leaving the home when agitated [4,7,10]. Those who leave home when agitated are generally in the presence of the caregiver at the point they leave the home but then become unsupervised when the caregiver does not accompany them as they leave. In this unaccompanied state, the PWD can fail to return home and require a search to be located.

#### 3.1.2. Situational Antecedents

Informal dementia caregivers living with PsWD report very high amounts of time in direct supervision, often indicating that 24-h supervision is required to prevent injuries and unintended exits [14,15,16]. Mahoney and colleagues [15] found that 52% of dementia caregivers felt they needed to be home with their care recipient 24 h a day; furthermore, 32.5% of caregivers reported they needed to be in the same room as the PWD 24 h a day. Since most caregiving is done by a single individual [17], this level of supervision is impossible on a daily basis, as caregivers must tend to personal needs, household responsibilities, and sleep. At these times, the PWD is reasonably and temporarily left unsupervised, a critical antecedent to most missing incidents.

Even if caregivers want to limit the time the PWD is unsupervised, affordable options may be limited. Often few or no other relatives live nearby. The cost of and difficulty in finding respite care, the lack of dementia-specific services (particularly in small or rural communities), and the difficulty in finding a reliable, affordable formal caregiver to provide in-home respite [18,19] makes it more likely the PWD is unsupervised at times, increasing the potential for a missing incident.

Missing incidents are often singular events with only a small percentage of PsWD having multiple incidents [4,6,7,20]. Furthermore, it is very difficult to predict when an event might occur. In a prospective, longitudinal study, it was discovered that most of the caregivers had not changed their routine prior to the missing episode, caregivers were in very close proximity most of the time and the PWD was involved in an everyday activity at the time of incident [4]. Additionally, in about 90% of the cases, no mental status changes were observed in the PWD prior to the missing incident.

Research on predictors of which PsWD will suffer a missing incident is limited. Reduced executive functioning and attention impairments [21], hippocampal lesions [12], and declining topographical memory [6] have all been associated with a higher risk of a missing incident. However, these are not factors generally known by caregivers, and to some extent occur in all PsWD. The lack of predictors in general, particularly observable ones, just prior to a missing incident, make prevention quite difficult.

For most caregivers, there often is not a clear definition of a missing incidents or an understanding of when their care recipient is actually at risk [22]. Seventy percent of caregivers believed that wandering and getting lost were behaviors that began in the later stages of the disease, and many differing terms were used by caregivers to describe the issue of missing incidents. This leaves caregivers in a precarious position of trying to simultaneously manage the independence and safety needs of the PWD without adequate tools for assessment or ability to predict problems [16].

#### 3.1.3. Neurocognitive Antecedents

A number of neurocognitive deficits occur as a result of the disease processes causing dementia that predispose a PWD to missing incidents and also contribute to the inability to return independently to home or to the caregiver. For instance, both declarative memory (remembering facts and events) and episodic memory (short term memory for recent events and contexts) are profoundly affected, even early in the disease. Both of these memory deficits can result in a missing incident as the PWD, who for instance has been instructed to wait in a specified location until the caregiver returns, forgets and walks away in a misguided attempt to find their caregiver [4]. Another critical deficit is anosognosia [23], or lack of insight, due to frontal lobe damage [24]. This damage is also associated with agitated and disinhibited behavior. Individuals with anosognosia are unaware of or underestimate their deficits and attempt unsafe activities such as leaving the home when agitated, walking or driving off alone when instructed to wait for the caregiver, or exiting the house into extreme weather conditions unprepared. Problems with impulsivity can also explain some of these behaviors and has been linked to propensity for missing incidents [25].

Executive function impairments are manifest as an alteration of personality, poor judgment, poor planning, difficulty with abstract reasoning, and an inability to complete complex tasks. Executive function deficits, in conjunction with attention impairments, have been associated with a greater likelihood of missing incident [21,25]. Mechanisms by which these impairments may lead to a missing incident include easy distractibility, poor wayfinding skills, and problems with complex tasks such as driving.

Disease-related changes to visuospatial and visuoperceptual processing are typically manifested as difficulty with navigation [26,27] and appear early in the disease process, particularly of Alzheimer’s disease [28]. Initially, navigation is impaired in unfamiliar locations, but later in the disease, PsWD have difficulty navigating even familiar surroundings. Visuospatial deficits include decreased ability to mentally rotate objects in which the same objects are unrecognized from different angles and PsWD prefer a frontal view [29]. Thus, if a PWD was driving a routine route but became distracted and drove past their destination, they might have a difficult time recognizing their destination when they turned around and approached the location from a different direction. Visuospatial deficits also effect topographical memory. Topographical memory involves the ability to recognize a familiar place or landmarks, orient oneself in space and recognize and follow a known route [30]. These deficits are present in even mild dementia with some worsening in moderate dementia.

PsWD may also exhibit visual agnosia (inability to recognize objects or places), even in familiar objects and places. For instance, a PWD who has lived in the same house for years can fail to recognize their surroundings and erroneously leave. Research suggests that scene recognition may become impaired earlier in the disease process, followed by face recognition, and may be a contributing factor to getting lost even early in the disease [31].

### 3.2. Missing Incident

In a confluence of the antecedent factors, plus currently unknown incident predictors, sometimes a PWD leaves a situation in which he or she was temporarily left alone. Often the primary caregiver was in very close proximity. What happens at this point in time is not reported in the research literature, but it is clear that some of these incidents become missing incidents in which the PWD’s location is unknown to the caregiver and a search is required to locate them. Findings from studies [1,6,9] on missing individuals can be summarized as: most incidents occur on foot during daylight hours; most are found quickly by the caregiver or someone in the neighborhood, but about a quarter require a formal search that involves law enforcement .Furthermore, missing incidents while driving are likely less than 5% of all incidents. These individuals are found farther away, and most are not driving at the point they are found [10].

### 3.3. Consequences

#### 3.3.1. Consequences to PWD

Missing incidents can cause consequences to the PWD during the time that they are missing, as well as subsequent to a successful return. Due to the neurocognitive deficits previously described, PWD do not correctly interpret the physiologic symptoms of thirst [32], and/or may not be able to execute a successful plan to obtain fluids safely, which may lead to dehydration, and death by exposure, if missing for an extended period [33]. Other adverse consequences include: minor injuries such as skin tears, which are generally due to falls; severe injuries such as fractures or dislocations; and death usually due to exposure [5,9,20,34,35]. Other causes of death include drowning and being hit by a vehicle while walking into an active roadway. Surprisingly, in four studies of missing incidents over time, none recorded incidents that ended in death [4,6,7,36]. This provides some clue to the very low incidence of death in all missing incidents. The longest study, which had up to a five-year follow-up, had a sample size of 104 individuals with a total of 201 incidents in which a PWD left a home unattended [6]. Two one-year longitudinal studies have also been conducted. In one study, 53 individuals were followed, with 23 unattended exits from home; in another study, 177 predominantly male veterans had 104 missing incidents [4,20]. In a Hong Kong retrospective study, there were no deaths in a sample of 251 PsWD, with 27.5% having at least one missing incident [7].

Subsequent to a successful return, negative consequences accrue, including increased risk of formal care placement and loss of independence [6]. PsWD who have a missing incident are seven times more likely than their counterparts to be placed in formal care [6]. For those PsWD who remain at home, both their independent activities and areas in which they can be unsupervised are curtailed [6,7]. While these are likely appropriate actions, they result in a reduced level of independence, and possibly reduced quality of life, for the PWD.

#### 3.3.2. Consequences to Caregiver

Caregivers for PsWD also experience consequences subsequent to a missing incident, including increased worry/psychological distress [7,37] and burden [38]. As previously mentioned, caregivers often cite the prevention strategy of increased vigilance to keep the PWD safe from unattended exits [22]. Nighttime is usually the most difficult period to sustain vigilance, and this causes the caregiver to awaken in an effort to determine the location of the PWD [39,40]. In a study of spousal caregivers of PsWD, 63% experienced sleep disruptions due to the nocturnal behavior of their care recipients [41]. Additionally, the generalized worry and burden caregivers experience contribute to the development and maintenance of sleep problems [42], with caregivers consistently reporting poorer sleep quality than non-caregivers [43]. In non-caregivers, inadequate sleep has been linked to depressive and anxiety disorders [44,45], functional decline [46], and other health problems [47,48]. Sustaining high levels of vigilance during the daytime can also take a toll on the caregiver. Between 44% and 60% of caregivers of PsWD are employed full or part-time [49], and most have work disruptions. A 2006 MetLife study of dementia caregivers in the workplace reported that about 70% arrive late, leave early, or take time off [50]. A third had to decrease the amount of hours they worked, 22% had to take a leave of absence, and 16% had to quit entirely [51]. Furthermore, caregivers for PsWD who exhibit behavior disturbances are more likely to reduce their hours at work [52].

## 4. Conclusions

Over a decade of research has provided the evidence used in the creation of this new conceptual model for understanding missing incidents in PsWD, a distinct behavioral symptom in dementia [1]. The intent of the provisional model is to highlight what is known, and what is as yet unknown, about the antecedents and consequences associated with these missing incidents. Ultimately, a final model can be used to help formal and informal caregivers differentiate missing incidents from wandering, and to develop effective prevention and recovery strategies.

Only by providing a detailed description of the contextual, situational and neurocognitive antecedents can caregivers focus specific strategies for prevention. While eyes-on supervision is the most important prevention strategy, it is not possible to provide this at the levels that are often needed. Unfortunately, little research could be found identifying other effective methods that caregivers use to prevent unattended exit from a safe situation, or to understand when independent trips should be limited or stopped. Strategies can be derived from the antecedent factors that are in play prior to an incident, such as greater family involvement in surveillance or use of formal care services instead of leaving the PWD unattended. Further research is needed to understand optimal prevention strategies and how to target these at the individual and contextual levels.

Another option for prevention that is frequently mentioned is the use of technologies. When presented with technology solutions, caregivers are rapid adopters. In a demonstration project, home-based primary care staff and caregivers of Veterans with dementia conducted an assessment of needs in the areas of ongoing surveillance, provision of care, prevention of injuries, and home safety, and identified technologies that might assist [53]. Initially, 60 Veterans were enrolled, and that data was used to create an algorithm. The algorithm assisted clinicians to assess problems that were potentially amenable to an available technology and identified targeted technologies. Both in this initial group, and when the program was implemented in practice, almost all caregivers adopted new technologies. In support of the high needs of caregivers around surveillance to prevent missing incidents, the primary products chosen were those that assisted caregivers in continuous surveillance of the care recipient including bed occupancy sensors, motion detectors and cameras with remote monitoring.

**Figure 2:**
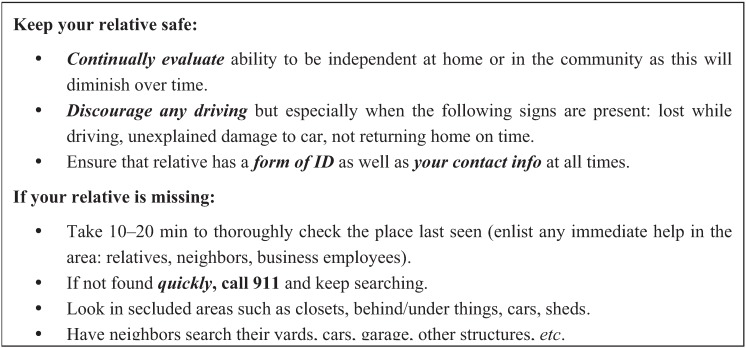
Missing incident plan magnet.

Another option, particularly when the PWD travels in the community alone, is tracking technology in which global positioning systems (GPS) and cellular phone signals are used in varying combinations to provide information on a tag’s whereabouts. Tag and the wearer’s location are usually referenced on a webpage-based map at the product’s website. Unfortunately, little research has been done to understand the best uses and keys to adoption of this technology [54].

The ultimate goal of this line of work is to gain a better understanding of the antecedents and other important factors that would better predict that a PWD is a greater or lesser risk of a missing incident. Enabling more independence can improve the quality of life for a PWD, but this can only be done when it is generally safe to do so. Future research is needed to test the accuracy and completeness of each concept, and the interconnections between the concepts. This work can be used to guide response strategies and policies, and training on those, for law enforcement agents [55] and caregivers of PsWD. In one example of how our current work is being used, our Consortium has assembled a list of preventative strategies and responses that we distribute to caregivers on a magnet (see Figure 2). Confirmation of the antecedents and interactions among the types of antecedents could assist caregivers in understanding the best response after a missing incident has occurred. Caregivers make few concrete changes after an incident, and further research spurred by this model may better inform healthcare providers to assist caregivers after an incident [7,11].
